# Casein Hydrolysate Alleviates Adipose Chronic Inflammation in High Fat-Diet Induced Obese C57BL/6J Mice through MAPK Pathway

**DOI:** 10.3390/nu15081813

**Published:** 2023-04-08

**Authors:** Ling Liu, Songfeng Yu, Tingting Bu, Guoqing He, Shanshan Li, Jianping Wu

**Affiliations:** 1 College of Biosystems Engineering and Food Science, Zhejiang University, 866 Yuhangtang Road, Hangzhou 310058, China; 2 Department of Food Science and Technology, Zhejiang University of Technology, Hangzhou 310014, China; 3 College of Animal Sciences, Zhejiang University, 866 Yuhangtang Road, Hangzhou 310058, China; 4 Department of Agricultural, Food and Nutritional Science, University of Alberta, Edmonton, AB T6G 2P5, Canada

**Keywords:** casein hydrolysate, adipose inflammation, NF-κB, MAPK-JNK, Erk

## Abstract

Obesity-induced adipose chronic inflammation is closely related to the development of insulin resistance and T2DM. Tripeptides l-valyl-l-prolyl-l-proline (VPP) and l-isoleucyl-l-prolyl-L-proline (IPP) derived from bovine casein have been reported to prevent inflammatory changes and mitigate insulin resistance in adipocytes. In this study, we aimed to investigate the influence of casein hydrolysates (CH) containing VPP and IPP on a high fat diet (HFD)-induced obese mice and cytokine TNF-α-induced adipocytes. Our data showed that CH alleviated chronic inflammation both in vivo and in vitro. 4% CH suppressed HFD-induced systemic inflammatory factors, hypertrophic white adipocytes, and macrophage infiltration. More importantly, CH was able to improve adipocyte dysfunction induced by TNF-α by increasing the expression of CCAAT/enhancer binding protein α (C/EBP-α) rather than peroxisome proliferator-activated receptor γ (PPAR-γ). Furthermore, CH also dose-dependently suppressed mitogen-activated protein kinase (MAPK)-c-Jun *N*-terminal kinase (JNK) phosphorylation and enhanced the phosphorylation of Erk 1/2, but not nuclear factor-kappa B (NF-κB) p65 phosphorylation, in TNF-α-induced 3T3-L1 cells. These results indicated that CH could ameliorate adipose chronic inflammation through the MAPK pathway. Altogether, our findings suggested that 4% CH supplementation for 6 weeks exerted a protective role in preventing obesity-related inflammation and adipose dysfunction.

## 1. Introduction

Obesity, characterized by excess adipose tissue mass expansion and fat accumulation, is a key contributor to the occurrence of insulin resistance and Type 2 diabetes mellitus (T2DM). The prevalence of T2D is increasingly rising worldwide and is expected to reach 18% in men and more than 21% in women by the year 2025 [1]. Obesity-induced insulin resistance is closely related to the development of other popular metabolic abnormalities including hypertension, dyslipidemia, hyperglycemia, hyperinsulinemia, coronary heart disease, hyperuricemia, non-alcoholic fatty liver disease as well as stroke [2]. Obesity is considered a state of low-grade chronic systemic inflammation especially adipose tissue inflammation [3], and is associated with the infiltration of inflammatory monocytes such as macrophages, in the expanding white adipose tissue, and secretion of pro-inflammatory cytokines such as tumor necrosis factor α (TNF-α), interleukin-1 β (IL-1β), interleukin-6 (IL-6), monocyte chemoattractant protein-1 (MCP-1), and among others [4]. Subsequently, more macrophages and immune cells are recruited by pro-inflammatory cytokines, leading to final adipocyte dysfunction and metabolic dysfunction, such as body glucose intolerance and insulin resistance [5]. Adipose tissue dysfunction is determined by impaired adipogenesis of the preadipocytes, altered lipid metabolism, adipocyte hypertrophy, and local inflammation [6]. This leads to ectopic fat deposition, resulting in increasing hypertrophic adipocytes and excess free circulating lipids accumulating in non-adipose organs such as the liver, heart, skeletal muscle as well as pancreas [7]. During this progression, multiple inflammatory signaling pathways including NF-κB and JNK pathways are activated, further contributing to the synthesis of more inflammatory cytokines and the development of systemic insulin resistance [8]. Pharmaceutical therapies in the treatment of obesity-related metabolic diseases are ineffective and associated with many side effects [9]. Therefore, intervention with alternative food-derived bioactive components is a promising natural approach to improve obesity-induced adipose tissue inflammation and insulin resistance [10].

Numerous food-derived bioactive ingredients including anthocyanins from purple corn [11], phenolic compounds in cocoa shells [12], lycopene [13], EPA and DHA [14], etc. showed potential in improving chronic inflammation and insulin resistance. Food-derived bioactive peptides were also shown to be beneficial against a variety of human health-threatening diseases, such as cardiovascular diseases, inflammation, and cancer [15]. They are encrypted within the primary structure of the parent proteins and remain inert, once released by hydrolysis, they will exert positive effects on the body [16]. As one of the high-quality natural protein sources for the human diet, bovine milk is considered to be a great source of bioactive peptides [17]. It has been reported that bovine α-lactalbumin hydrolysate could ameliorate HFD-induced adipose tissue insulin resistance and inflammation via inhibitor of kappa B kinase (IKK) and MAPK signaling pathways in C57BL/6J mice [18]. Casein is the major protein in bovine milk, accounting for about 83.85% of total protein. The most well-known casein-derived peptides are angiotensin converting enzyme (ACE)-inhibitory tripeptides VPP and IPP, both of which were originally identified from fermented milk with a starter containing *Lactobacillus helveticus* and *Saccharomyces cerevisiae* [19], and exhibited blood pressure-lowering effect in both humans and animals [20,21]. Currently, the anti-hypertensive activity and the possible mechanism of casein hydrolysates containing VPP and IPP have been comprehensively reviewed [22,23]. In addition, casein hydrolysates containing VPP and IPP also improved vascular endothelial function [24]. Furthermore, long-term intake of casein hydrolysates containing these antihypertensive peptides could also impart cardiovascular benefits [25]. Aihara et al. [26] and Sawada et al. [27] demonstrated that oral administration of bovine milk casein-derived peptide VPP could attenuate obesity-induced adipose inflammation in mice. However, whether casein hydrolysates containing VPP/IPP would play a beneficial role in HFD-induced chronic adipose tissue inflammation is not known. In this study, an *Aspergillus oryzae*-derived protease called “Amano” 2SD (A2SD) was employed to release VPP/IPP from casein. The anti-inflammatory effect of bovine CH on the adipose tissue of HFD-fed mice and the underlying molecular mechanism via 3T3-L1 adipocytes impaired by pro-inflammatory cytokine TNF-α were investigated. Our work showed that bovine CH improved adipose dysfunction and inhibited chronic inflammation likely through MAPK-dependent manner.

## 2. Materials and Methods

### 2.1. Reagents and Materials

The BCA protein assay kit and radio-immunoprecipitation assay (RIPA) lysis buffer were purchased from Beyotime Biotechnology (Shanghai, China). The primary antibodies, anti-α/β-tubulin (cat. #2148), anti-IκBα (cat. #9242), anti-PPAR-γ (cat. #2430), anti-C/EBPα (cat. #2295), anti-NF-κB p65 (cat. #8242), anti-p-NF-κB p65 (cat. #3033), anti-Erk 1/2/p-Erk 1/2 (cat. #8201) and anti-p-JNK (cat. #4668) were purchased from Cell Signaling Technology (Beverly, MA, USA). The primary antibodies against JNK were obtained from Beyotime Biotechnology (Shanghai, China). The secondary antibodies including anti-mouse and anti-rabbit immunoglobulin G (IgG) antibody were purchased from Licor Biosciences (Lincoln, NE, USA). The ACCU-CHEK blood glucose test strips and glucometer were from Roche (Mannheim, Germany). The ultrasensitive mouse insulin enzyme-linked immunosorbent assay (ELISA) kit was purchased from Nanjing Jiancheng Biotechnology Research Institute (Nanjing, China) and Elabscience (Wuhan, China).

### 2.2. Preparation of CH and Determination of VPP and IPP

Milk casein was purchased from Changzhou Shuyishuer biotechnology Co., Ltd. (Changzhou, China). The purity of the casein was 88%. The Casein was hydrolyzed by Protease A “Amano” 2SD from Amano Seisakusho Co., Ltd. (Shanghai, China) at a concentration of 6% (*w*/*v*). The enzyme/substrate was 2:100 (*w*/*w*). The hydrolysis condition was performed at 50 °C and pH 7.0. After 5 h, the casein hydrolysates were boiled for 15 min to deactivate the enzyme, and then centrifuged at 10,000× *g* for 10 min at 4 °C. The supernatant was collected and lyophilized for subsequent diet preparation. The protein proportion of CH powder was determined by Kjeldahl. O-phthaldialdehyde (OPA) assay was used to determine the degree of hydrolysis of CH. The value of the degree of hydrolysis was measured as 32.31%. The quantification of VPP and IPP was performed as our previous work with HPLC mobile phase modification [28], namely, 5% B, 1 min; 5–40% B, 1–8 min; 40–95% B, 8–12 min; 95% B, 12–14 min. The flow rate and temperature of gradient elution for samples were 0.3 mL/min and 40 °C, respectively. The two peptides were determined and quantified by liquid chromatography (LC)-MS/MS with multiple reaction monitoring (MRM) through a triple quadrupole mass spectrometer (Agilent 6460, Agilent Technologies, Santa Clara, CA, USA).

### 2.3. Animal Experiments and Diet

All operational procedures involving animal usage were approved by the Medical Ethics Committee of Experimental Animal Care at Zhejiang Chinese Medical University (Approval code: 10214). Four-week-old male C57BL/6J mice (SPF, *n* = 36) were purchased from Shanghai SLAC Laboratory Animal Co., Ltd. (Shanghai, China) and kept in a controlled environment at 23 ± 1 °C and 50–60% relative humidity, 12/12 h light/dark cycle (lights were on during 8:00–20:00) with food and water ad libitum. All mice were raised four mice per cage. Twelve mice were randomly selected and fed a normal chow diet (16.5% kcal from fat, 3712 kcal/kg), and the others were fed a high-fat diet (HFD, provided 60% kcal from fat, 5242.88 kcal/kg) for 7 weeks to build an obesity mice model. Animals that were fed HFD were subsequently randomly divided into two groups which would be assigned to two trials: HFD, HFD + 4% CH (4 g/100 g HFD) (*n* = 12 for each group). Our preliminary experiment showed that neither 0.05% nor 2% CH had positive activities in improving glucose intolerance in HFD-fed mice. Thus, 4% CH was adopted in this study. The compositions of the chow diet, HFD, and HFD supplemented with 4% CH were shown in supporting information (Table S1). All diets were stored at 4 °C in a dark room before use. During the experiment, the body weight of each mouse was monitored once a week, and food intake was recorded twice a week. At the end of the experiment, all mice were sacrificed after a 16 h overnight fasting. Blood samples of mice were collected carefully and centrifuged at 3000× *g* for 10 min to obtain the supernatant serum. Organ and tissues including liver, kidney, muscle, epididymal, brown scapular, subcutaneous, perirenal and mesentery fat pads were dissected and weighted, all organ and tissue samples were collected and stored at −80 °C for further analysis.

### 2.4. Oral Glucose Tolerance Test (OGTT) and Intraperitoneal Insulin Tolerance Test (IPITT)

An oral glucose tolerance test of all mice was carried out at the end of the fifth week of 4% CH supplementation. The mice were fasted overnight (16 h) with a subsequent oral administration of glucose at 2.0 g/kg body weight. The blood was sampled from the tail vein and determined with blood glucose test strips and glucometer at first (t = 0), and 15, 30, 60, 90, and 120 min, respectively, after intragastric glucose administration. Three days later following the OGTT, all mice underwent an intraperitoneal injection of recombinant human insulin (0.75 IU/kg body weight) after a 6-h fasting period. The blood glucose of mice were determined at the same time points as OGTT. After plotting the glucose concentration curve against the time, the corresponding areas under the curve (AUC) for blood glucose were calculated.

### 2.5. Plasma Glucose, Insulin, Lipids, and Homeostasis Model Assessment of Insulin Resistance (HOMA-IR)

Fasting glucose, fasting insulin, total cholesterol (TC), triglyceride (TG), high-density lipoprotein-cholesterol (HDL-C), low-density lipoprotein-cholesterol (LDL-C), nonesterified fatty acid (NEFA), IL-6, IL1-β, MCP-1, and TNF-α were measured with the commercially available enzymatic assay kits according to the manufacturer’s instructions. The HOMA-IR index was calculated as the equation: HOMA-IR = fasting serum glucose (mmol/L) × fasting serum insulin (μU/mL)/22.5 [29].

### 2.6. Cell Culture and Adipocyte Inflammation Induction

3T3-L1 fibroblasts, bought from the cell bank of the Chinese Academy of Sciences, Shanghai, were revived and cultured as previously methods by Zebisch et al. with slight modifications [30]. That is, Dulbecco’s modified Eagle’s medium (DMEM) supplemented with 10% fetal bovine serum (FBS) and 1% antibiotics was prepared for cell growth. The 3T3-L1 were cultured at 37 °C with 5% CO_2_ in a humidified atmosphere. The cells were subcultured into a 48-well plate at a density of 1 × 10^4^ cells/well. When confluence reached more than 90%, then continue to grow for another 48 h. Subsequently, the growth medium was replaced with a differentiation medium, DMEM supplemented with 10% FBS, 1 µM dexamethasone (Dex), 0.5 mM 3-isobutyl-1-methylxanthine (IBMX), and 1% antibiotics, to induce differentiation. At the same time, 0.5, 1, and 2.5 mg/mL CH was added respectively. After 3T3-L1 cells were incubated with CH for 1 h, 10 ng/mL TNF-α was added to stimulate inflammation for 24 h, then the medium was renewed after 48 h and continue to culture for another 3 days. At the end of the differentiation process, lipid staining with Oil Red O was performed. Ten percent formalin was used to fix the cells for 1 h. A working solution of Oil Red O (0.3% prepared with isopropanol) was then added to each well for 1 h in a lucifuge place. Pictures were taken with an Olympus microscope CKX53 (Olympus, Tokyo, Japan).

### 2.7. Western Blot Analysis

3T3-L1 were seeded at a density of 2 × 10^5^ cells/well in the 6 well plates. CH intervention and TNF-α induced inflammation was performed at the aforementioned dosage. At the end of the experiment, 150 μL RIPA lysis buffer was added to each well. Cells were collected and centrifuged at a speed of 12,000× *g* for 15 min at 4 °C. The supernatant protein extracts were quantified with a BCA protein assay kit. After adjusting the protein concentration to the same amount with loading buffer and denaturing the protein in boiling water for 5 min, aliquots of protein samples were subjected to 10% sodium dodecyl sulfate polyacrylamide gel electrophoresis (SDS-PAGE), and the protein bands were then transferred to polyvinylidene fluoride (PVDF) membranes. The bands were incubated with Odyssey Blocking Buffer for 1 h with gentle shaking, followed by the incubation of diluted primary antibody including anti-tubulin, anti-PPAR-γ, anti-C/EBPα, anti-IκBα, anti-p-65, anti-p-p65, anti-JNK, anti-p-JNK, anti-Erk(1/2) and anti-p-Erk(1/2) overnight at 4 °C. The blots were then washed 4 times (each for 5 min) with TBST, followed by incubation in diluted labeled fluorescent-labeled secondary antibodies at room temperature with gentle shaking for 1 h. Membranes should be protected from light during incubation. After four 5-min washes with TBS-T, blots were rinsed with TBS to remove residual Tween 20 and the bands were then scanned and analyzed by Licor Odyssey Clx system (Licor Biosciences, Lincoln, NE, USA) and Image Studio Ver 5.2, respectively.

### 2.8. Morphological and Histopathological Analysis of Liver and Epidydimal Adipose Tissue

Fresh liver and epididymal adipose tissue samples were fixed in 4% paraformaldehyde and then embedded in paraffin, the dissected sections were stained with haematoxylin and eosin (HE) for morphological observations. Images were captured using Olympus IX81 fluorescent microscope (Markham, ON, Canada) and presented at 100× magnification. Immunohistochemistry staining was conducted with F4/80 primary antibody (Cell Signaling Technology, Beverly, MA, USA) to analyze the macrophage infiltration in adipose tissue.

### 2.9. Statistical Analysis

The results were analyzed using SPSS 22.0 (IBM Inc., Chicago, IL, USA) by one-way analysis of variance (ANOVA) and Duncan’s multiple-comparison test and presented as mean ± standard deviation (SD) of at least three repetitions. *p* < 0.05 was considered to be statistically significant and expressed as letters (different superscript letters indicate statistical significance). The graph was created on sigma plot 12.5 (San Jose, CA, USA).

## 3. Results

### 3.1. Detection and Quantification of Antihypertensive Tripeptides in Casein Hydrolysates

**Figure 1:**
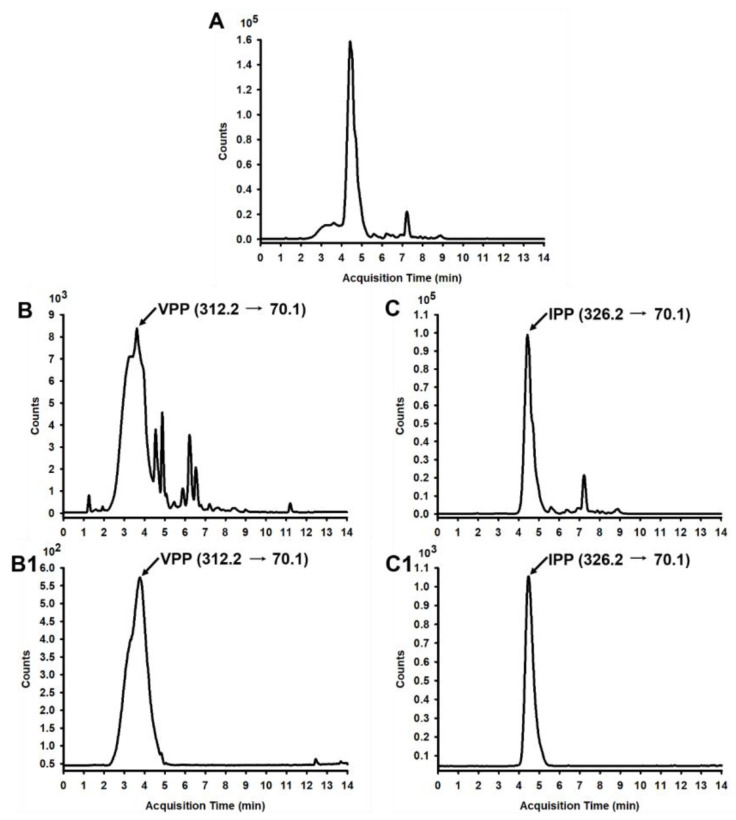
LC-MS/MS chromatogram of VPP and IPP in CH. (**A**) TIC of CH; (**B**) Chromatogram of VPP in CH in positive MRM mode, and the retention time (RT) of VPP is 3.500 min; (**B1**) Standard chromatogram (SC) of VPP in positive MRM mode; (**C**) Chromatogram of IPP in CH in positive MRM mode, and the RT of IPP is 4.968 min; (**C1**) SC of IPP in positive MRM mode.

An *Aspergillus oryzae*-derived protease called Sumizyme FP was previously shown to release successfully short and proline-rich antihypertensive peptides from casein among several commercially available proteases [31]. Firstly, we determined if VPP and IPP could be released enzymatically from casein by another *Aspergillus oryzae*-derived Protease A “Amano” 2SD. Next, LC-MS/MS analysis was performed to quantify these two peptides in casein hydrolysates. Both the two tripeptides VPP and IPP were detected, and the total ion chromatogram (TIC) of casein hydrolysates was shown in Figure 1A. Figure 1B,C were the chromatograms of VPP and IPP in positive MRM mode; Figure 1B1,C1 were respective peptide standards. A content of VPP 184.93 ± 1.32 µg/g and IPP 926.69 ± 1.58 µg/g dry basis were obtained, respectively. The protein content of the CH sample was determined as 82.87%.

### 3.2. Effect of CH Containing Antihypertensive Peptides on the Physiological Parameters of HFD-Induced Obesity Mice

**Figure 2:**
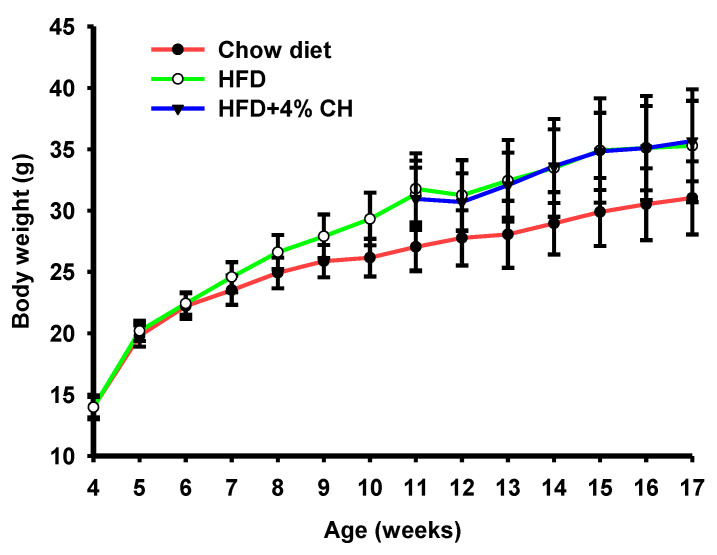
The effects of CH supplementation on the body weight change of obese mice induced by HFD.

To investigate the effect of CH on obesity-induced insulin resistance and adipocyte inflammation, C57BL/6J mice were fed either a chow diet or HFD for 7 weeks, followed by supplementation with vehicle or 4% CH for another 6 weeks. The physiological parameters of each treatment group was shown in Table 1. The energy intake, liver, kidney, muscle, mesenteric and brown fat weight did not differ between groups. Compared with the mice of the chow diet-fed group, the HFD-fed mice had significantly increased epididymal fat, subcutaneous fat, and perinephric fat. However, no significant differences were observed between the HFD group and the CH treatment group (Table 1). According to the growth curve of mice, the body weight of HFD-fed mice was obviously increased. After supplementation with CH for six weeks, there was no significant differentiation in the body weight gain of mice between the HFD and HFD + 4% CH group (Figure 2).

### 3.3. CH Ameliorated HFD-Induced Insulin Intolerance and Hyperinsulinemia in HFD-Fed C57BL/6J Mice

**Figure 3:**
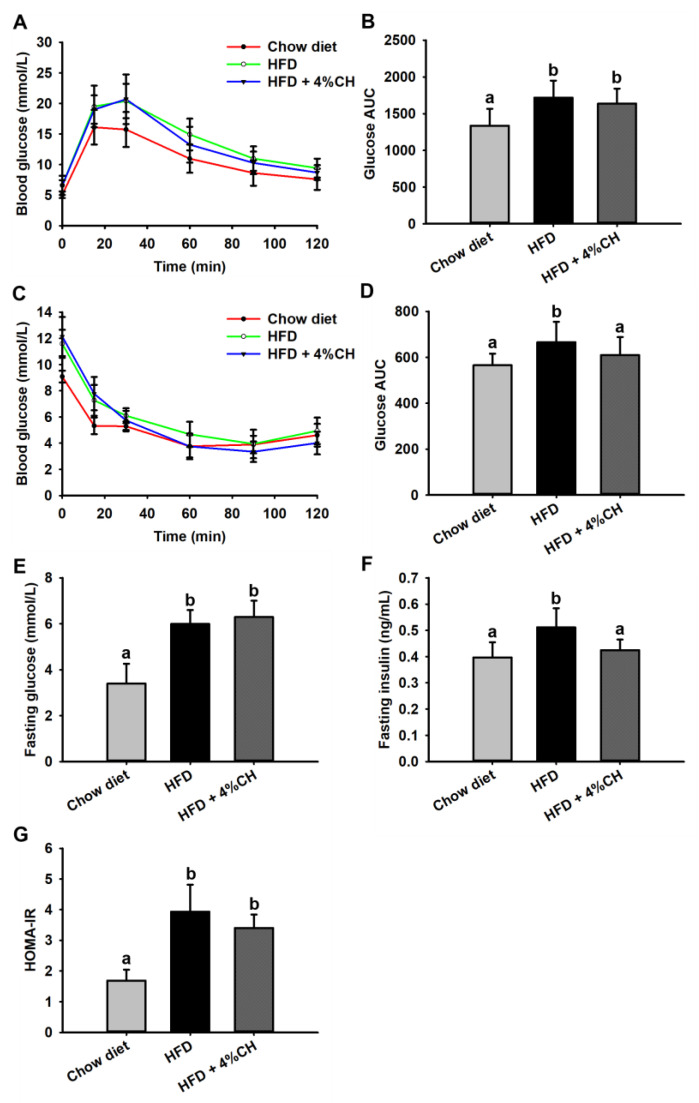
The effects of CH supplementation on the blood glucose tolerance and insulin tolerance of mice fed with HFD. The curve of blood glucose level at different time intervals in OGTT assay (**A**) and the AUC value of blood glucose curve in OGTT assay (**B**), the curve of blood glucose level at different time intervals in IPITT assay (**C**) and the AUC value of blood glucose curve in IPITT assay (**D**), fasting glucose level (**E**), fasting insulin level (**F**) and HOMA-IR (**G**), all values were exhibited as means ± SD (*n* = 12). Different letters indicated statistically significant difference at *p* < 0.05.

Effect of CH administration on HFD-induced glucose intolerance and insulin intolerance was investigated. HFD caused a significant decrease in glucose tolerance, in comparison with the chow diet group (Figure 3A); however, no difference was observed in the AUC value of the OGTT test between the mice of the HFD group and the HFD + 4% CH group (Figure 3B). Compared with the mice fed with a chow diet, the insulin tolerance of mice fed with HFD was significantly decreased (Figure 3C); supplementation with 4% CH significantly ameliorated insulin intolerance (*p* < 0.05) (Figure 3D), indicating the ability of CH in improving the insulin sensitivity of mice in HFD group. Similarly, supplementation with 4% CH did not affect the fasting glucose (Figure 3E), whereas supplementation with 4% CH significantly reduced the fasting insulin, compared with the HFD group (Figure 3F). Intriguingly, The HOMA-IR, calculated from the fasting plasma insulin and fasting plasma glucose, was reduced in comparison with the HFD group, even though the difference was not significant (Figure 3G). These results suggested that CH could partially improve insulin resistance in mice induced by HFD.

### 3.4. Effects of CH Containing Antihypertensive Peptides on the Plasma Lipid Metabolism Parameters of HFD-Induced Obese Mice

**Figure 4:**
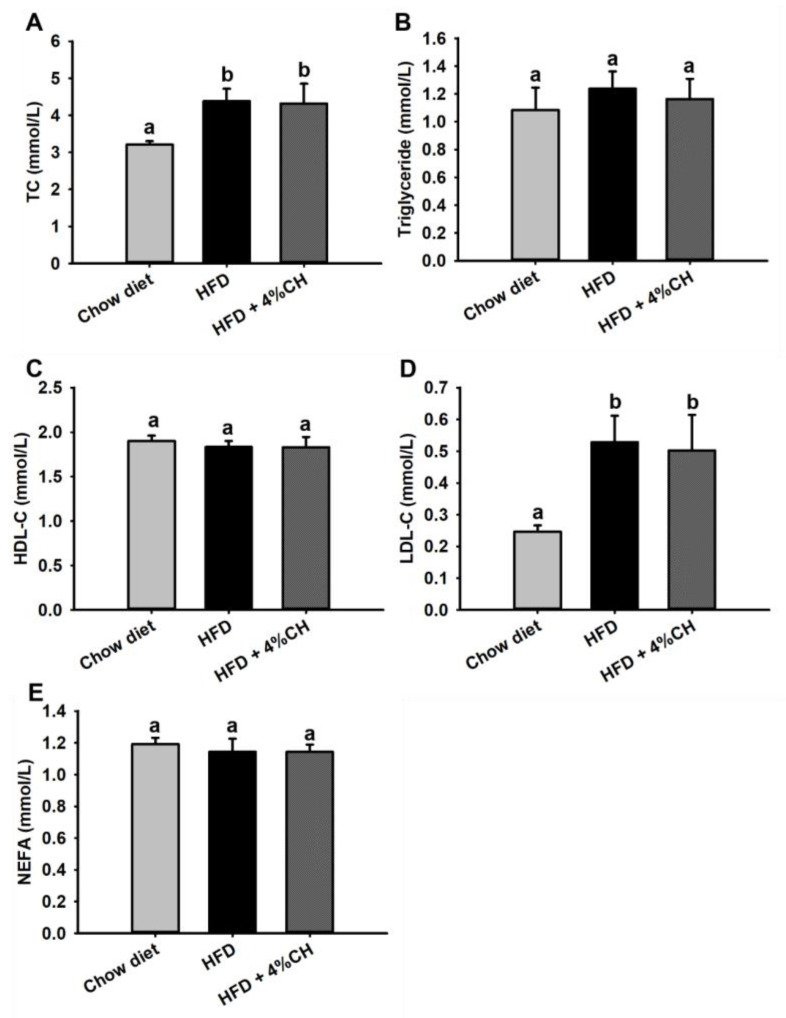
The effects of CH on the plasma lipid profile in HFD-induced obesity mice. The levels of TC (**A**), triglyceride (**B**), HDL-C (**C**), LDL-C (**D**) and NEFA (**E**) in the serum of mice. All data were exhibited as means ± SD (*n* = 12). Different superscript letters in each column indicated a statistically significant difference at *p* < 0.05.

The plasma lipid metabolism profile was further investigated after animal experiments. Compared with the Chow diet group, HFD consumption induced significant increased TC and LDL-C levels in obese mice as shown in Figure 4A,D. Supplementation with 4% CH didn’t reverse this increase. At the same time, no significant differences were observed in triglyceride, HDL-C and NEFA levels among each treatment group (Figure 4A,C,E). These data indicated that lipid metabolism was not affected by CH supplementation.

### 3.5. CH Alleviated Chronic Inflammation in HFD-Induced Obese Mice

**Figure 5:**
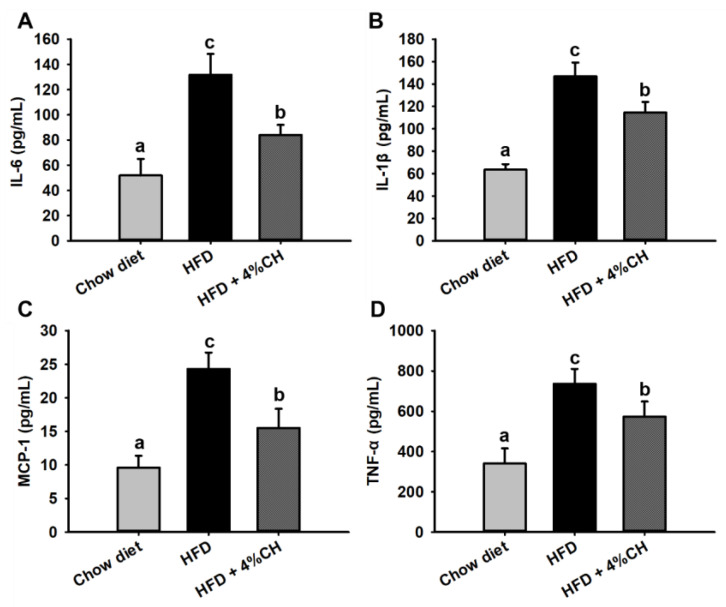
The effects of CH supplementation on the serum inflammatory factors in HFD-induced obese mice. The level of IL-6 (**A**), IL-1β (**B**), MCP-1 (**C**) and TNF-α (**D**) in the serum of mice. All data were shown as means ± SD (*n* = 12). Data sharing the different superscript letters in each column indicated that the difference between each group was statistically significant at *p* < 0.05.

The levels of cytokine inflammatory factors were determined to investigate the effect of CH administration on chronic inflammation in HFD-induced obese mice. As shown in Figure 5, pro-inflammatory cytokines IL-6 (Figure 5A), IL-1β (Figure 5B), MCP-1 (Figure 5C) and TNF-α (Figure 5D) levels in HFD-fed mice were all markedly augmented compared to the chow diet group. All these inflammatory cytokines in obese mice were significantly suppressed after supplementation with CH (Figure 5, *p* < 0.05). These results suggested that CH could significantly reduce inflammatory cytokines level and improve chronic inflammation in HFD-induced obese mice.

### 3.6. Effect of CH Supplementation on the Epididymal Adipose and Liver Tissues in HFD-Induced Obese Mice

**Figure 6:**
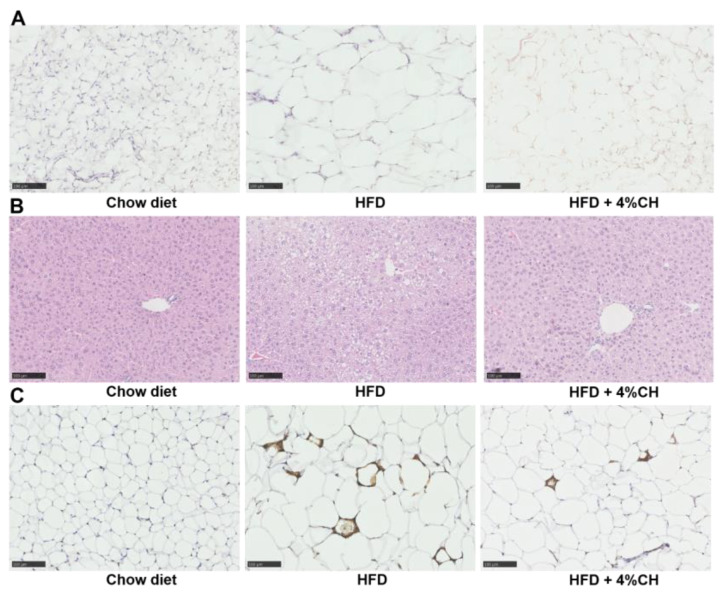
The effects of CH consumption on the histological change of epididymal adipose and liver tissue. Representative sections of epididymal white adipose tissue (**A**) and liver (**B**) stained with HE. Representative adipose tissue sections immunostained with F4/80 primary antibody (**C**). All images were presented at 100× magnification.

To investigate the effect of CH supplementation on the histological alteration and macrophage infiltration, HE staining and immunohistochemistry staining were performed to assess the morphological change and macrophage accumulation. The staining results were shown in Figure 6. In comparison with the chow diet mice, HFD-induced mice were observed to have large amounts of hypertrophic adipocytes (Figure 6A) and lipid vacuoles (Figure 6B) in adipose tissue and liver tissue, respectively. These pathological alterations were alleviated after supplementation with 4% CH (Figure 6A,B). Furthermore, many crown-like structures, which were macrophages marked with F4/80 (a macrophage-specific surface marker) primary antibody, were observed surrounding the hypertrophic adipocytes (Figure 6C). Thus, HFD caused macrophage infiltration in white adipose tissue such as epididymal adipose in obese mice, whereas such inflammatory infiltration was attenuated after CH supplementation (Figure 6C). This result was consistent with Figure 5 and demonstrated that CH treatment was effective in ameliorating chronic inflammation in HFD-fed mice.

### 3.7. CH Improved the Adipocyte Dysfunction Induced by TNF-α

**Figure 7:**
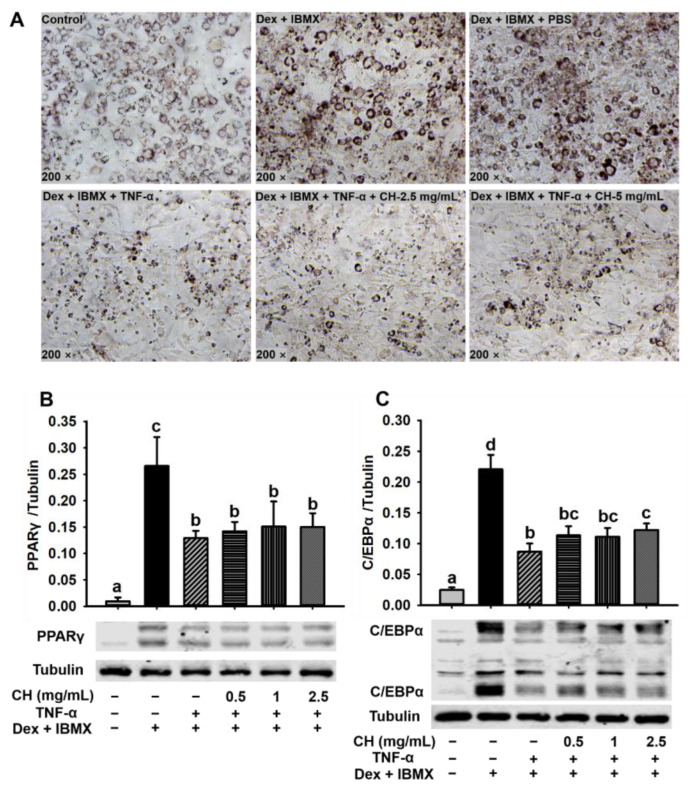
CH alleviated impaired adipocyte differentiation induced by TNF-α. 3T3-L1 were incubated in a differentiation medium containing 2.5 mg/mL and 5 mg/mL CH for 1 h, followed by the treatment of 10 ng/mL TNF-α for 24 h, and then refreshed with a new differentiation medium with or without CH after 48 h and 72 h, respectively. Oil red O staining was used to detect the accumulation of lipid droplets during cell differentiation (**A**). 3T3-L1 cells were seeded in 6-well plates, and incubated in differentiation medium containing 0.5, 1 and 2.5 mg/mL CH for 1 h, respectively. Then 10 ng/mL TNF-α was added and incubated for 24 h. Then, new differentiation medium containing the corresponding CH were refreshed and cultured for another 48 h and 72 h. The expression levels of PPAR-γ (**B**) and C/EBPα (**C**) were detected by western blot analysis. Different superscript letters in each column indicated statistically significant difference at *p* < 0.05.

The inflammatory model of 3T3-L1 preadipocyte differentiation was applied to assess the molecular mechanism of CH to ameliorate chronic inflammation in HFD-induced mice. Differentiation of 3T3-L1 preadipocytes was induced by Dex and IBMX for 6 days. As shown in Figure 7A, cells in the control group did not differentiate, while cells supplemented with Dex and IBMX were differentiated normally into a large amount of ring lipid droplets. The degree of differentiation of 3T3-L1 showed no difference in the Dex and IBMX treatment with or without PBS. Namely, PBS solvent did not affect the normal differentiation of 3T3-L1 preadipocytes. However, 3T3-L1 preadipocyte differentiation was impaired after being treated with 10 ng/mL TNF-α for 24 h (Figure 7A). In the Dex + IBMX + TNF-α group, the cells failed to differentiate and the abnormal cells were contracted and crumpled into small spots after staining with oil red O. In contrast, after treatment with 2.5 and 5 mg/mL CH, the differentiation dysfunction of 3T3-L1 preadipocytes was significantly improved. Cell malformation was relieved to some extent and a small number of ring lipid droplets started to reappear (Figure 7A). Next, we determined the protein expression of two important key preadipocyte differentiation regulators PPAR-γ and C/EBPα. Figure 7B,C showed that the protein expression of PPAR-γ and C/EBPα were significantly increased in 3T3-L1 supplemented with Dex and IBMX compared with the control group. Whereas, after stimulation by pro-inflammatory cytokine TNF-α, the levels of PPAR-γ and C/EBPα in 3T3-L1 were significantly decreased. Although the treatment with 0.5, 1 and 2.5 mg/mL CH didn’t reverse the expression downregulation of PPAR-γ, the decreased expression of C/EBP-α tended to be restored after CH supplementation. Meanwhile, CH treatment with a concentration of 2.5 mg/mL in 3T3-L1 significantly elevated the expression of C/EBP-α (*p* < 0.05, Figure 7C). These results indicated that C/EBP-α was a potential therapeutic target for CH to ameliorate inflammatory cytokine-induced impaired adipogenesis.

### 3.8. Effects of CH on the NF-κB and MAPK Signal Pathway in Impaired 3T3-L1 Cells

**Figure 8:**
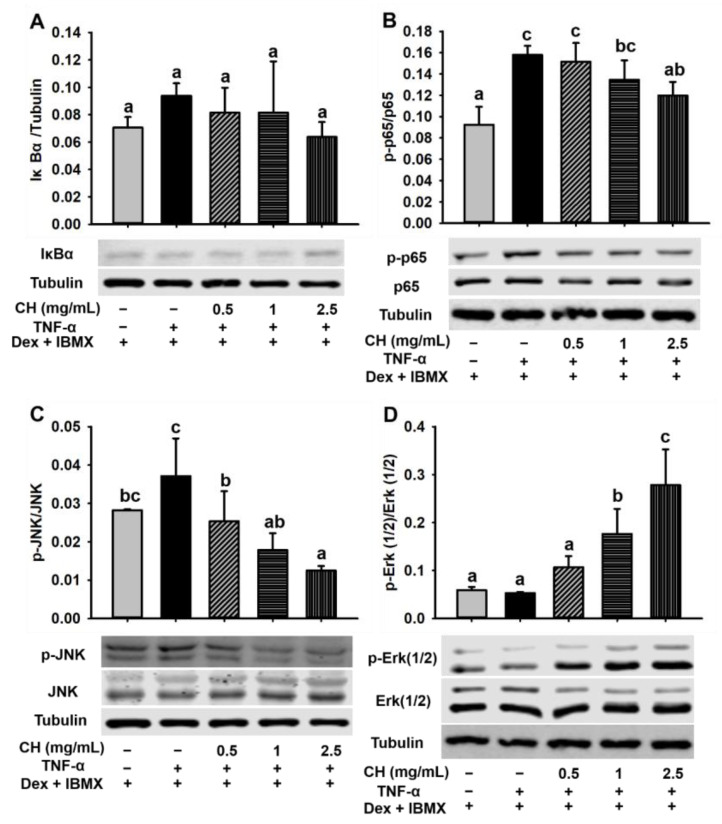
CH improved the adipocyte inflammation induced by TNF-α via the mediation of NF-κB p65 and MAPK signal pathway. 3T3-L1 cells were seeded in 6-well plates, and preincubated in differentiation medium containing 0.5, 1 and 2.5 mg/mL CH for 1 h, respectively. Then 10 ng/mL TNF-α was added and incubated for 24 h. Then, new differentiation medium containing the corresponding CH was replaced and cultured for another 48 h and 72 h. Western blot was used to detect the expression levels of IκBα (**A**), p-p65 (**B**), p-JNK (**C**), and p-Erk 1/2 (**D**). Different superscript letters meant a statistically significant difference at *p* < 0.05.

The results of differentiation dysfunction of 3T3-L1 preadipocytes induced by TNF-α suggested that cell shrinkage was due to a chronic inflammatory response. In order to further explore the mechanism of CH treatment in the improvement of TNF-α-induced inflammation in 3T3-L1 cells, we investigated the effect of CH on the NF-κB and MAPK signaling pathway related to adipocyte inflammation. In light of the critical negative role of IκBα (an inhibitor of NF-κB) and p65 activation in proinflammatory factor-mediated NF-κB signaling pathway in adipocyte inflammation, we hypothesized that CH might exert a protective effect on the activation of NF-κB pathway. Thus, IκBα expression and p65 phosphorylation in impaired 3T3-L1 cells were detected (Figure 8A,B). As expected, p-p65 level was greatly augmented due to TNF-α stimulation but was remarkedly rescued by 2.5 mg/mL CH supplementation (*p* < 0.05) (Figure 8B). Interestingly, the content of IκBα did not differ among each group (*p* > 0.05) (Figure 8A) In contrast, the phosphorylation of JNK and Erk 1/2 in the MAPK pathway was dose-dependently affected by the treatment with CH. As revealed in the Figure 8C,D, CH supplementation at the concentrations of 0.5, 1 and 2.5 mg/mL in impaired 3T3-L1 cells suppressed the upregulated p-JNK induced by TNF-α in a dose-dependent way. With the rise of the concentration of CH, the phosphorylation level of JNK decreased gradually. Compared with the cells stimulated by TNF-α, the inhibition of p-JNK during adipose dysfunction was significant at all concentrations tested (*p* < 0.05, Figure 8C). Furthermore, Figure 8D showed that the expression of p-Erk 1/2 was not significantly altered after TNF-α addition, whereas, as the concentration of CH went from 0.5 to 2.5 mg/mL, the expression of p-Erk 1/2 was elevated in a dose-dependent way, especially at the CH concentrations of 1 and 2.5 mg/mL, the phosphorylation of Erk 1/2 was significantly increased (*p* < 0.05, Figure 8D). These results suggested that CH improved TNF-α-induced chronic inflammation in adipocytes mainly via regulating the MAPK signaling pathway.

## 4. Discussion

Obesity, caused by excess nutrients and energy intake, is strongly associated with insulin resistance and T2DM in modern life [32]. It is well-accepted that obesity results in low-grade, chronic inflammation in adipose tissue, which leads to the final pathogenesis of insulin resistance [33]. In our recent study, we investigated the effects of casein hydrolysates containing tripeptides VPP and IPP on the chronic inflammation induced by HFD in C57BL/6J mice. Casein-derived VPP and IPP were reported to induce beneficial adipogenic differentiation and improve inflammatory changes in 3T3-F442A adipocytes [34]. Both peptides could also enhance insulin signaling and mitigated insulin resistance in TNF-α-stressed preadipocytes [35]. Moreover, oral administration of VPP alone exerted an obvious anti-inflammatory effect and inhibited adipose inflammation on the adipose tissue of HFD-induced mice and high-fat high-sucrose-fed mice, respectively [26,27]. However, whether casein hydrolysates containing VPP and IPP exhibit a protective and therapeutic effect on obesity-associated insulin resistance and adipose inflammation is still unknown. Therefore, we first try to release VPP or IPP encrypted in casein by *Aspergillus oryzae*-derived protease “A2SD”, which was reported to be able to release ACE inhibitory peptides rich in proline [31]. LC-MS/MS analysis in positive MRM mode was used to detect and quantify these two peptides. Similarly, Matsuura et al. [36] also prepared a powdered CH containing VPP and IPP with *Aspergillus oryzae* protease. In our preliminary experiment, CH supplementation with 0.05% and 2% were applied to the HFD-induced obese mice, whereas, neither 0.05% nor 2% CH had positive activities in improving the glucose intolerance in HFD-fed mice. We speculated that increasing the dosage of CH may be effective in ameliorating HFD-induced insulin resistance. A previous study reported that 4% egg white hydrolysates could significantly enhance insulin sensitivity in HFD-induced insulin-resistant rats [37]. In this study, 4% CH was applied to investigate the anti-insulin-resistant and anti-inflammatory activity of CH-containing VPP and IPP in HFD-fed mice. The results showed that neither the body weight gain nor visceral adipose including epididymal fat, subcutaneous fat, and perinephric fat of HFD-induced obese mice could be significantly reduced after intervention with CH (Table 1). As shown in Figure 1, CH administration had no effect on the body weight increase of HFD-fed mice, which concluded that CH was not suitable to be used to lose weight in HFD-induced obesity. In line with this result, intervention with synthetic tripeptide VPP alone could also not prevent the progression of normality to obesity even though feeding VPP from the start of the experiment [27]. Additionally, feeding 4% CH containing VPP and IPP could significantly improve the insulin intolerance in obese mice induced by HFD but had no obvious effect on glucose intolerance as shown in the results of OGTT and IPITT results in Figure 3B,D. Similarly, Liu et al. reported that jiaogulan tea-derived gypenosides could also only reduce insulin intolerance rather than glucose intolerance in IPITT and OGTT tests of HFD-fed mice, respectively [38]. However, Different from our results, another CH was reported to protect mice against HFD-induced glucose intolerance only, but not insulin intolerance [10], which was probably due to the difference of CH fed to mice. Meanwhile, serum insulin levels rather than glucose levels were observed to be decreased in response to CH supplementation (Figure 3E,F). It is likely that CH ingestion contributes to ameliorating hyperinsulinemia in obese mice induced by HFD. CH tended to have a protective potential on HFD-induced insulin resistance as shown in Figure 3G. However, how CH improved insulin signal remains to be further investigated.

We next measured the serum lipids and inflammatory cytokines to evaluate the systemic lipid metabolism dysfunction and inflammation in the HFD group and HFD + 4% CH group. The CH supplementation did not effectively alleviate the high TC and LDL-cholesterol level in obese mice induced by HFD (*p* > 0.05, Figure 4), indicating that CH had no adverse effect on the systemic lipid level either in the Chow diet group or the HFD group. Proinflammatory mediators such as TNF-α, IL-6, and IL-1β played pivotal roles in the development of insulin resistance in adipose tissue of HFD-fed obese mice [39]. HFD could activate monocytes and M1-type macrophages to accumulate in visceral fat and increase the contents of proinflammatory cytokines MCP-1, TNF-α, IL-1β, and IL-6 in adipose tissue. Moreover, MCP-1 further recruited more macrophages into adipose tissue and exacerbated adipose tissue inflammation [40]. In our study, the serum levels of adipocytokines IL-6, IL-1β, TNF-α, and MCP-1 in the CH treatment group were all significantly lower when compared to those in the HFD group (*p* < 0.05, Figure 5A–D). Therefore, we infer that CH may exert anti-inflammatory activities against adipose chronic inflammation induced by HFD and provide a protective role in adipocyte dysfunction in obese mice. Adipose tissue is primarily composed of adipocytes, the main function of which is to store excess lipids in the body [41]. Due to the long-term HFD administration, the caloric balance was disrupted and the new adipogenesis from preadipocytes was impaired, which led to the excessive hypertrophy of fat cells. Consequently, adipocyte dysfunction and adipose tissue endocrine and immune response were elicited [42]. As expected, our results showed that 4% CH supplementation successfully reversed the over-expansion of epididymal adipocytes in obese mice (Figure 6A). Alone with the beneficial effects on the adipocytes, 4% CH also prevented the hepatic steatosis induced by HFD. As shown in Figure 6B, the lipid inclusions and hepatocellular vacuoles were obviously reduced after CH supplementation compared to the HFD-induced mice. Furthermore, the infiltration of proinflammatory M1 macrophages into the epididymal adipose tissue was dramatically decreased in the HFD + 4% CH group indicated by the reduced numbers of crown-like structure (Figure 6C). Taken together, to the best of our knowledge, this study is the first to report that CH containing VPP and IPP play a prominent role in alleviating HFD-induced adipocytes dysfunction and chronic inflammation in the white adipose tissue of obese mice, although the specific mechanism remains to be further elucidated.

It has been reported that persistent low-grade and chronic inflammation in adipocytes ultimately impaired preadipocyte differentiation [43]. In order to explore the mechanism of CH to improve adipocyte dysfunction and inflammation, impaired 3T3-L1 cells induced by inflammatory cytokine TNF-α was established. Consistent with previous results [43], adipocyte differentiation was disrupted and hindered in the presence of TNF-α (Figure 7A). The ring lipid drops disappeared and cells contracted following the stimulation with TNF-α, which was in agreement with previous studies that the capacity of adipocytes to store lipids was decreased due to TNF-α [44]. Nonetheless, the accumulation of lipid droplets started to recover partially and the inflammatory state was improved to some extent following CH treatment (Figure 7A). C/EBP-α and PPAR-γ were two key transcription factors involved in preadipocyte differentiation. The expressions of these two proteins were upregulated during the normal preadipocyte differentiation [45]. Therefore, we hypothesized that CH may improve the impaired adipocytes differentiation by upregulating the expression of transcription factors C/EBP-α and PPAR-γ. Our data showed that C/EBP-α and PPAR-γ were both dramatically upregulated under normal differentiation (Figure 7B,C), which was in accordance with a previous report [34]. After being stimulated by TNF-α, the expression levels were all significantly decreased. However, no significant differences were observed in the expression levels of PPAR-γ following CH treatment (Figure 7B). In contrast, CH supplementation at a concentration of 2.5 mg/mL significantly restored the downregulation of C/EBP-α (*p* < 0.05, Figure 7C). CH treatment with light concentrations at 0.5 and 1 mg/mL also upregulated the expression C/EBP-α, although they were not statistically significant (*p* > 0.05, Figure 7C). All these results in this study revealed for the first time that CH was likely to improve the TNF-α-induced adipocyte dysfunction by upregulating the expression of C/EBP-α.

Inflammatory cytokine TNF-α mediates adipocyte inflammation through a series of signaling cascades. NF-κB pathway and MAPK pathway are the two main signaling pathways for chronic inflammation [46]. The NF-κB signaling pathway is a classic inflammatory regulatory system that can be activated under the stimulation of LPS and TNF-α. NF-κB is a homodimer consisting of p65 and p50. Under normal curcumstances, NF-κB (p65/p50) is inhibited by inhibitory protein IκBα. Once activated, IκBα is phosphorylated and subsequently degraded by ubiquitination proteasome. The released NF-κB then translocates to the nucleus to initiate inflammation by mediating the transcription and translation of inflammatory cytokines [47]. MAPK signaling pathway is mainly involved in three serine/threonine protein kinases including Erk 1/2, p38 and JNK. A variety of cellular activities including proliferation, differentiation, survival, apoptosis, transformation, and immune response are associated with this pathway [48]. In this study, our data revealed a prominent role for CH in preventing TNF-α-induced phosphorylation of NF-κB p65 (*p* < 0.05, Figure 8B). Intriguingly, TNF-α-induced degradation of IκBα molecules was not affected (Figure 8A), which was similar to previous research that an egg-derived tripeptide prevented p65 translocation into the nucleus of endothelial cells without affecting the degradation of IκBα [49]. Therefore, we speculated that peptides having anti-inflammatory activities may inhibit NF-κB by regulating the expression of the downstream protein of IκBα. In addition, by analyzing the two key proteins JNK and Erk1/2 in MAPK pathway, we found that the phosphorylation of JNK was dramatically negatively correlated with the concentration of CH from 0.5 to 2.5 mg/mL (Figure 8C), whereas the phosphorylation level of the extracellular regulated kinase (Erk 1/2) was significantly positively elevated with the rising of CH concentration (Figure 8D). These results indicated that both the inhibition of JNK phosphorylation and upregulation of Erk 1/2 phosphorylation by CH treatment were in a dose-dependent way. It’s reported that the signaling cascade leading to the activation of the Erk signaling pathway was mainly involved in cell proliferation, transformation, and differentiation. In contrast, JNK- and p38-MAPK pathways were mainly involved in apoptosis, stress, and inflammatory responses [50]. Therefore, the inhibition of MAPK-JNK and activation of MAPK-Erk signaling pathway by CH may be related to its effect on preventing inflammation of 3T3-L1 adipocytes impaired by TNF-α and promoting normal differentiation of adipocytes, respectively. The specific peptides in CH contributing to the beneficial effects of CH in ameliorating the chronic inflammation of HFD-fed mice are as yet incompletely understood. Previous research suggested that VPP could be fully absorbed into the intestine and then into the systemic circulation [51,52]. Therefore, VPP and IPP may enter the microvessels of adipose tissue and play an anti-inflammatory role. It can be inferred that the anti-inflammatory effects of CH may be due to the presence of anti-inflammatory peptides including VPP and IPP. The subsequent separation and identification of these functional peptides and illuminating their anti-inflammatory mechanism are of great importance in future research.

Casein is the major protein in bovine milk. CH prepared by different proteases has been extensively used in functional food products. This study investigated the anti-inflammatory activity of CH systematically and illustrated the possible anti-inflammatory mechanism. However, the specific anti-inflammatory peptides remain to be separated and identified. Since allergenic peptides would be possibly generated due to the mild enzyme hydrolysis, the potential allergenicity of CH should be assessed to ensure the safety of CH peptide products. Additionally, more efforts should be taken to study the bioavailability and metabolic stability of CH to improve the practical application value in the future.

## 5. Conclusions

In summary, our findings suggested that CH suppressed chronic inflammation both in adipose tissue of HFD-induced obese mice and cytokines-mediated impaired adipocytes. The underlying mechanism of CH is likely via the MAPK pathway. Given the critical roles of adipose chronic inflammation in the pathogenesis of obesity-induced insulin resistance and T2DM, CH may serve as a functional food product to exert a protective and therapeutic role in the management of obesity-related diseases and complications.

## Figures and Tables

**Table 1:** The effect of CH on food intake, body weight change, and organ weights in C57BL/6J mice.

Food Intake, Body Weight, Organ Weight	Chow Diet	HFD	HFD + 4% CH
Initial body weight (g)	27.05 ± 2.00 ^a^	31.77 ± 2.89 ^b^	30.96 ± 2.55 ^b^
Final body weight (g)	31.03 ± 2.98 ^a^	35.29 ± 4.60 ^b^	35.67 ± 3.28 ^b^
Body weight change (g)	3.26 ± 1.35 ^a^	4.04 ± 2.41 ^b^	4.97 ± 2.05 ^b^
Food intake (g)	3.42 ± 0.32 ^a^	2.36 ± 0.17 ^b^	2.32 ± 0.17 ^b^
Energy intake (g)	12.69 ± 1.20 ^a^	12.37 ± 0.89 ^a^	12.17 ± 0.92 ^a^
Organ weight (g)			
Liver	0.99 ± 0.11 ^a^	0.98 ± 0.14 ^a^	1.03 ± 0.15 ^a^
Kidney	0.32 ± 0.03 ^a^	0.36 ± 0.03 ^a^	0.34 ± 0.02 ^a^
Muscle	0.35 ± 0.10 ^a^	0.38 ± 0.09 ^a^	0.37 ± 0.05 ^a^
Epididymal fat	0.74 ± 0.21 ^a^	1.28 ± 0.44 ^b^	1.24 ± 0.42 ^b^
Mesenteric fat	0.23 ± 0.11 ^a^	0.27 ± 0.13 ^a^	0.30 ± 0.11 ^a^
Brown fat	0.12 ± 0.05 ^a^	0.14 ± 0.06 ^a^	0.12 ± 0.04 ^a^
Subcutaneous fat	0.35 ± 0.11 ^a^	0.76 ± 0.54 ^b^	0.75 ± 0.38 ^b^
Perinephric fat	0.23 ± 0.08 ^a^	0.48 ± 0.21 ^b^	0.49 ± 0.22 ^b^

Note: Different superscript letters meant significant differences at *p* < 0.05.

## Supplementary Materials

The following supporting information can be downloaded at: https://www.mdpi.com/article/10.3390/nu15081813/s1, Table S1: The composition and calorie supplementation of high-fat diet and casein hydrolysates-containing diet fed to mice.
