# Supplementary material for: Casein Hydrolysate Alleviates Adipose Chronic Inflammation in High Fat-Diet Induced Obese C57BL/6J Mice through MAPK Pathway
Source: Nutrients. 2023 Apr 8;15(8):1813. doi: 10.3390/nu15081813 (PMC10146021; doi:10.3390/nu15081813)
Supplement: Supplementary file 1 [file nutrients-15-01813-s001.zip › nutrients-2314348-supplementary.pdf]

Table S1 The composition and calories supplementation of high fat diet and casein hydrolysates-containing diet fed to mice

	HFD		HFD + 4%CH	
Main component	gm%	kcal%	gm%	kcal%
Protein	26	20	26	20
Carbohydrate	26	20	26	20
Fat	35	60	35	60
Total		100		100
kcal/gm	5242.88		5242.88	
Ingredient	gm	kcal	gm	kcal
Casein, 88%	258.45	1033.79	221.18	1033.79
Casein hydrolysates	0.00	0.00	40	
L-Cystine	3.88	15.51	3.88	15.51
Maltodextrin	161.53	646.12	161.53	646.12
Sucrose	88.91	355.62	88.91	355.62
Cellulose	64.61	0.00	61.88	0.00
Soybean Oil	32.31	290.75	32.31	290.75
Lard	316.60	2849.39	316.60	2849.39
Mineral Mix S10026	12.92	0.00	12.92	0.00
DiCalcium Phosphate	16.80	0.00	16.80	0.00
Calcium Carbonate	7.11	0.00	7.11	0.00
Potassium Citrate, 1 H_2O	21.32	0.00	21.32	0.00
Vitamin Mix V10001	12.92	51.69	12.92	51.69
Choline Bitartrate	2.58	0.00	2.58	0.00
